# A microbiota-bile acid-immune axis orchestrates intestinal antiviral defense: CDCA-to-UDCA biotransformation by *Limosilactobacillus reuteri* LRM2293 is associated with PEDV resistance

**DOI:** 10.1186/s12917-026-05928-4

**Published:** 2026-09-22

**Authors:** Ling Sui, Qing Xiang, Yuning Kou, Jiaxuan Li, Yanping Jiang, Wen Cui, Weichun Xie, Xiaona Wang, Lijie Tang

**Affiliations:** 1 College of Veterinary Medicine, Northeast Agricultural University, Harbin, 150030 China; 2 Heilongjiang Key Laboratory for Animal Disease Control and Pharmaceutical Development, Harbin, 150030 China

**Keywords:** *Limosilactobacillus reuteri*, PEDV, Bile acid, UDCA, Antiviral activity

## Abstract

Porcine epidemic diarrhea virus (PEDV) causes severe intestinal injury and high mortality in neonatal piglets, and effective immunomodulatory strategies remain limited. Here, we investigate a probiotic-derived metabolic axis associated with resistance to PEDV infection through bile acid–immune crosstalk. A *Limosilactobacillus reuteri* strain (LRM2293), isolated from disease-resistant Min pigs, efficiently converted CDCA into ursodeoxycholic acid (UDCA). Both LRM2293 and UDCA significantly reduced PEDV replication in intestinal epithelial cells and ameliorated clinical symptoms in neonatal piglets. UDCA treatment increased type III interferon (IFN-λ3) expression, while pharmacological inhibition of TGR5 partially reversed both the antiviral effects of UDCA and IFN-λ3 induction. These findings support the potential involvement of TGR5-associated IFN-λ3 signaling in UDCA-mediated antiviral activity. In addition, UDCA preserved epithelial barrier integrity and attenuated oxidative stress, inflammatory responses, and endoplasmic reticulum stress in infected intestines.

Collectively, our findings suggest that LRM2293-mediated bile acid remodeling is associated with enhanced resistance to PEDV infection. TGR5-associated IFN-λ3 signaling may represent one mechanism contributing to the antiviral effects of UDCA.

## Introduction

Porcine epidemic diarrhea virus (PEDV) causes severe diarrhea, dehydration, and elevated mortality rates in neonatal piglets, resulting in substantial economic losses to the global swine industry [1–3]. PEDV primarily targets intestinal epithelial cells and induces villous atrophy, disruption of epithelial barrier integrity, excessive inflammatory responses, oxidative stress, and endoplasmic reticulum (ER) stress, ultimately leading to intestinal dysfunction and impaired nutrient absorption [4–7]. Due to the continuous emergence of PEDV variants and the limited duration of protective mucosal immunity induced by current vaccines, the development of alternative intervention strategies remains an important research priority [8–10].

Min pigs, an indigenous Chinese breed, exhibit superior disease resistance and environmental adaptability in comparison to commercial Duroc × Landrace × Yorkshire (DLY) pigs [11–14]. Previous studies have demonstrated significant differences in gut microbial composition, diversity, and metabolic functions between Min pigs and commercial breeds [15, 16]. These observations suggested that specific microbial populations and their metabolites may contribute to the enhanced disease resistance phenotype observed in Min pigs. Consequently, identifying and characterizing beneficial microorganisms derived from Min pigs may provide new opportunities for improving host resistance against enteric diseases.

Probiotics serve as a promising complementary strategy for the prevention and control of enteric viral infections through modulation of intestinal homeostasis and host immune responses. *Limosilactobacillus reuteri* (*L. reuteri*) is an important intestinal probiotic that contributes to intestinal and hepatic homeostasis, regulates immune responses, improves metabolic health, and protects against pathogen infection [17–20]. In addition, *L. reuteri* participates in bile acid metabolism through enzymes such as bile salt hydrolase (BSH), thereby influencing bile acid composition and host-microbe interactions [21, 22]. However, substantial strain-dependent variation exists in bile acid metabolic capacity, and whether probiotic-mediated bile acid biotransformation contributes to antiviral immunity against PEDV remains poorly understood. The *L. reuteri* strain LRM2293 (accession number PQ451445.1) was isolated from Min pigs’ intestines. LRM2293 shows promising probiotic traits such as effective bile acid transformation, enhanced UDCA production, protection against intestinal barrier damage, and inflammation regulation [15]. These features indicated that LRM2293 could be a unique probiotic strain with potential antiviral benefits.

Bile acids are signaling molecules that aid in lipid digestion and impact metabolism, barrier function, inflammation, and immunity [23–25]. Gut microbiota can transform primary bile acids into various secondary bile acids [24, 25]. Our research shows that gut microbiota of Min pigs effectively converts primary bile acids into ursodeoxycholic acid (UDCA) via 7α-hydroxysteroid dehydrogenase (7α-HSDH) and 7β-hydroxysteroid dehydrogenase (7β-HSDH) [15, 26]. UDCA is recognized for its anti-inflammatory, antioxidant, and cell-protective benefits, stress relief, and potential antiviral properties [27, 28]. The role of microbiota-derived UDCA in resisting PEDV infection is still unclear. Recent studies suggest that the G protein-coupled bile acid receptor TGR5 plays a role in antiviral immunity by regulating interferon responses in the gut [29]. TGR5 activation is linked to increased interferon production and resistance to intestinal viral infections [30]. However, the involvement of bile acid-mediated immune pathways in PEDV infection remains to be fully understood.

Therefore, we hypothesized that Min pig-derived *L. reuteri* strains may possess enhanced antiviral activity associated with bile acid remodeling and increased UDCA production. This study comprehensively assessed the bile acid biotransformation capacities and antiviral activities of *L. reuteri* strains isolated from Min pigs and DLY pigs. We further investigated the potential involvement of TGR5-associated interferon signaling and evaluated the protective effects of LRM2293 and UDCA in a neonatal piglet PEDV challenge model. Collectively, this study provides new insight into the link between probiotic-mediated bile acid metabolism and antiviral immunity and highlights a potential microbiota–bile acid–immune axis involved in host resistance to PEDV infection.

## Materials and methods

### Bacterial strains, cell, virus, and reagents

Pig-derived *L. reuteri* strains, including Min strains (LRM2293, AM13, AM255) and DLY *L. reuteri* strains (CO21, J31, J32), were preserved in our laboratory [15]. All strains were cultured in MRS broth. IPI-2I cells, obtained from BNCC Biotechnology Co., Ltd. (Beijing, China), were selected due to their porcine intestinal epithelial origin and susceptibility to PEDV, providing a biologically relevant in vitro model for studying PEDV–host interactions [31]. PEDV HEB strain was preserved in our laboratory [13]. Ursodeoxycholic acid (UDCA; HPLC ≥ 98%, catalog no. B21405; Shanghai Yuanye Bio-Technology Co., Ltd., Shanghai, China) and chenodeoxycholic acid (CDCA; ≥98%, catalog no. C804610; Shanghai Macklin Biochemical Co., Ltd., Shanghai, China) were used in this study. UDCA and CDCA were dissolved in DMSO as stock solutions, and the final concentration of DMSO did not exceed 0.1% (v/v) in all cell-based assays.

### Determination of 7α-HSDH and 7β-HSDH activities

The activities of 7α-HSDH and 7β-HSDH in *L. reuteri* strains were quantified using commercially available enzyme-linked immunosorbent assay (ELISA) kits (Wuhan Enzyme Immunoassay Co., Ltd., Wuhan, China) according to the manufacturer’s instructions. Strains were incubated with 200µM CDCA at 37 °C for 24 h then centrifuged to collect supernatant for enzyme analysis.

For intracellular enzyme extraction, bacterial pellets were washed twice with sterile PBS and lysed by ultrasonic disruption on ice. Protein concentrations were determined using a BCA Protein Assay Kit (Thermo Fisher Scientific, USA). Enzyme levels were normalized to total protein concentrations and expressed as relative enzyme activity units.

### LC-MS analysis of CDCA biotransformation

To evaluate bile acid biotransformation capacity, *L. reuteri* strains LRM2293 and J31 were cultured in MRS medium containing 200 µM CDCA at 37 °C for 24 h. After incubation, culture supernatants were collected by centrifugation (4,000 rpm, 10 min) and analyzed using LC-MS [32]. LC-MS data acquisition and further analyses were conducted by Shanghai Biotree Biotech Co., Ltd. UDCA concentrations were quantified using authentic analytical standards, and metabolite identification was performed based on retention time and mass spectral matching. LRM2293 (Min pig-derived) and J31 (DLY pig-derived) were used to represent relatively high- and low-CDCA-to-UDCA conversion phenotypes, respectively, for subsequent comparative analyses. Basal MRS controls with and without CDCA were not included; therefore, the reported UDCA values represent measured concentrations without baseline subtraction.

### Cytotoxicity assay (CCK-8)

The cytotoxicity was assessed using a Cell Counting Kit-8 (CCK-8; Dojindo, Kumamoto, Japan) assay [31]. In brief, IPI-2I cells were treated with serial dilutions of CDCA-transformed bacterial supernatants, UDCA (0-100 µM), or SBI-115 (0–50 µM) for 24 h. After incubation with 10 µL of CCK-8 for 1 h at 37 °C, absorbance at 450 nm was measured using a Bio-Rad microplate reader. Cell viability was calculated as a percentage of the untreated control: Cell viability (%) = (OD₄₅₀ treatment / OD₄₅₀ control) × 100. The maximum non-cytotoxic concentration was the highest level that kept cell viability over 80%. Experiments were performed using three independent biological replicates, with each treatment tested in triplicate wells. The final concentration of DMSO (vehicle) did not exceed 0.1% (v/v) in all treatment groups, and the vehicle control was included in every assay.

### Anti-PEDV activity of CDCA-transformed supernatants from *L. reuteri* strains

The antiviral effects of CDCA-transformed supernatants from various L. reuteri strains were tested in IPI-2I cells. An MOI (multiplicity of infection) of 0.1 was selected based on previously established PEDV infection models in IPI-2I cells [33], which yields robust viral replication while allowing evaluation of antiviral interventions over a 24 h period. After reaching 80–90% confluence, cells were infected with PEDV HEB at an MOI of 0.1 for 1 h at 37 °C. Unbound virus was washed off, and cells were treated with non-cytotoxic CDCA supernatant dilutions for 24 h. Virus control groups received only culture medium, while uninfected cells served as negative controls. Post-incubation, viral RNA levels were quantified by RT-qPCR, and infectious viral titers were determined by plaque assays. Indirect immunofluorescence assay (IFA) was performed to detect the expression of PEDV S protein and evaluate viral infection at the cellular level.

### Anti-PEDV activity assay of UDCA in vitro

**Fig. 1:**
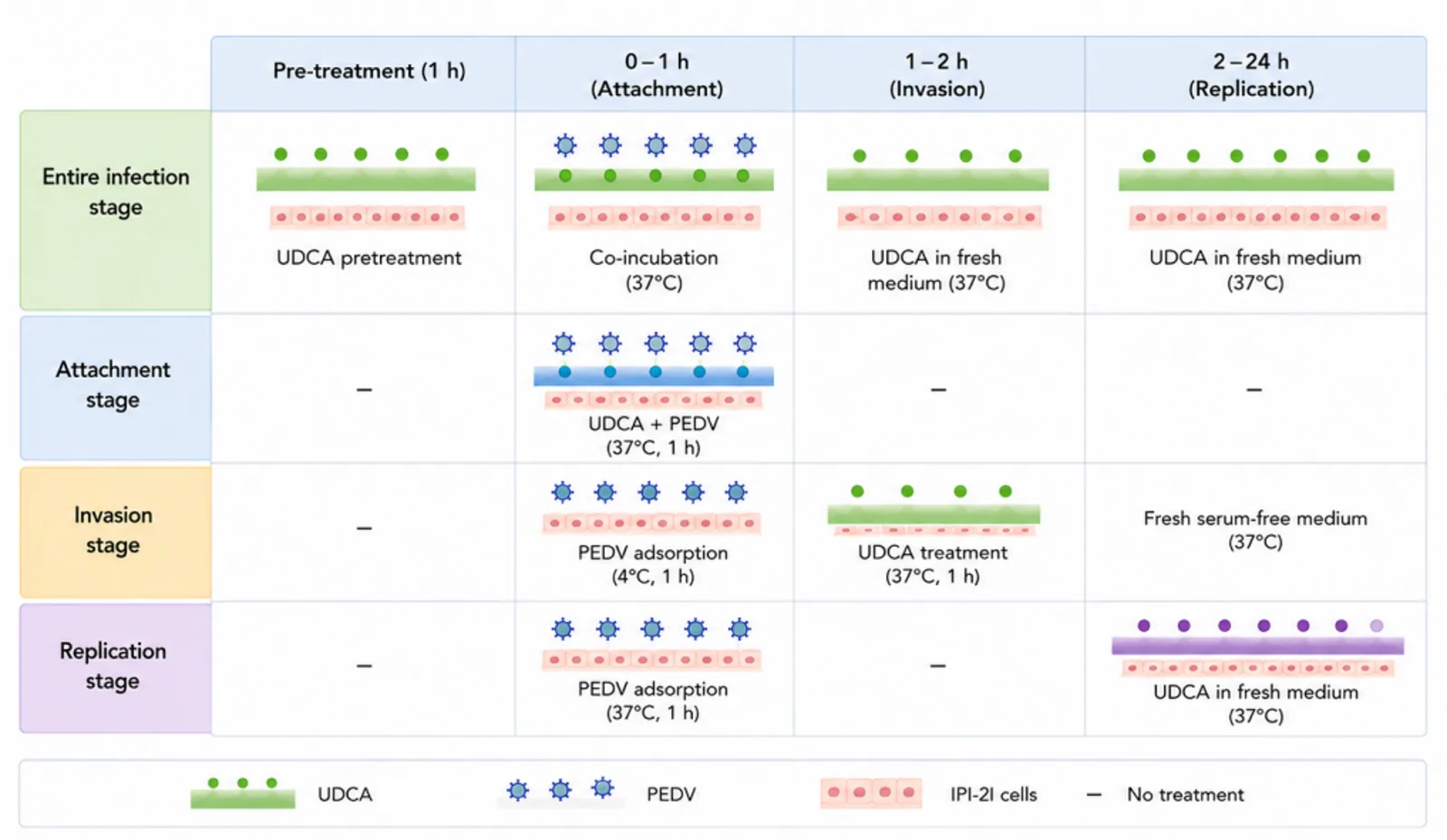
Experimental scheme for stage-specific UDCA treatment during PEDV infection. UDCA treatment was administered during different stages of the PEDV life cycle, including viral attachment (0–1 h), invasion (1–2 h), replication (2–24 h), or throughout the entire infection period. The initial flowchart sketch was generated with ChatGPT-4o (OpenAI), and the authors revised all content, logic and layout to finalize the figure

To investigate the stage-specific antiviral activity of UDCA, treatment was performed during different phases of the PEDV infection cycle, including viral attachment, invasion, replication, and the entire infection period (Fig. 1). Following treatment, supernatants and cell lysates were analyzed using RT-qPCR, plaque assays, and IFA to evaluate PEDV infection.

### RT-qPCR analysis

Total RNA was extracted utilizing the TRIzol reagent according to the manufacturer’s instructions. cDNA synthesis was performed using a commercial reverse transcription kit (Takara, Dalian, China). Quantitative real-time PCR was performed using SYBR Premix Ex Taq II on a Roche LightCycler 480 system. β-actin was used as the reference gene for normalization. Relative gene expression levels were calculated using the 2^^−ΔΔCt^ method. All primers used for PEDV-N and host genes were designed in this study using Primer-BLAST (NCBI), and the corresponding primer sequences are provided in Table 1.

**Table 1:** Primer sequences

Targets	PCR Primer ( 5’-3’ )	Amplicon Length (bp)
PEDV-N	F: GGTATTGGAGAAAATCCTGACAGGCATAAGCAACAGCA	249
	R: GACGCATCAACACCTTTTTCGACAAATTCCGCATC	
Claudin-1	F: AGATTTACTCCTACGCTGGTGAC	194
	R: GCAAAGTGGTGTTCAGATTCAG	
Occludin	F: GTAGTCGGGTTCGTTTCC	146
	R: GACCTGATTGCCTAGAGTGT	
ZO-1	F: GAGGCTCAGCCCTATCCATCTG	218
	R: CGGGACCTGCTCATAACTTCGT	
β-catenin	F: GCTGCTGTTTTGTTCCGAATGTC	173
	R: CCTGGGCACCAATATCAAGTCC	
E-cadherin	F: CCCCAACACTTCTCCCTTCACT	129
	R: CTCGAGGGTTTTCTTTGGCTTC	
IL-1β	F: GCCTTCAGTCCAGTCGCCTTCT	162
	R: GTTGTCACCATTGTTAGCCATCAC	
IL-18	F: GCTGCCATGTCAGAAGACTCA	205
	R: TCCGGGGTGCATTATCTCTAC	
TNF-α	F: CACCACGCTCTTCTGCCTAC	181
	R: GGCTTTGACATTGGCTACAACG	
GRP78	F: GCTCTGGAAATGGAAACAGC	154
	R: TCGTCCCACAGATACCATCC	
CHOP	F: ATCTCATCCCCAGGAAACGAAG	137
	R: AACTCTGACTGGAATCTGGAGAGC	

### Plaque assay

To determine infectious PEDV titers, plaque assays were performed as follows. Briefly, IPI-2I cells were infected with PEDV and cultured under an agarose overlay consisting of 2× MEM and 2% low-melting-point agarose (1:1, v/v) supplemented with 6 µg/mL trypsin. After incubation at 37 °C for 48 h, cells were fixed with 4% paraformaldehyde overnight at 4 °C and stained with 0.2% crystal violet. Plaques were counted manually, and viral titers were expressed as plaque-forming units per milliliter (PFU/mL). Plaques were counted manually by two independent investigators in a blinded manner, and counts were cross-validated; discrepancies were resolved by consensus re-counting.

### Indirect immunofluorescence assay (IFA)

Cells were fixed using 4% paraformaldehyde (Tianjin Alphabio Co., Ltd., Tianjin, China) and subsequently permeabilized with 0.1% Triton X-100 (Elabscience Biotechnology Co., Ltd., Wuhan, China). Blocking was performed with 5% BSA. The anti-PEDV antibody generated by our laboratory was used at a dilution of 1:100, and the GRP78 primary antibody (catalog no. YT5858, ImmunoWay, USA) was used at a dilution of 1:300. Both primary antibodies were incubated overnight at 4 °C. The secondary antibodies, goat anti-rabbit IgG (H + L), F(ab′)₂ fragment (Alexa Fluor^®^ 488 conjugate, catalog no. 4412 S; Alexa Fluor^®^ 594 conjugate, catalog no. 8889 S; Cell Signaling Technology, USA), were used at a dilution of 1:500 and incubated for 1 h at room temperature in the dark. Nuclei were counterstained with DAPI (catalog no. 4083 S, Cell Signaling Technology, 1:5000).

### Animal experiment design

**Fig. 2:**
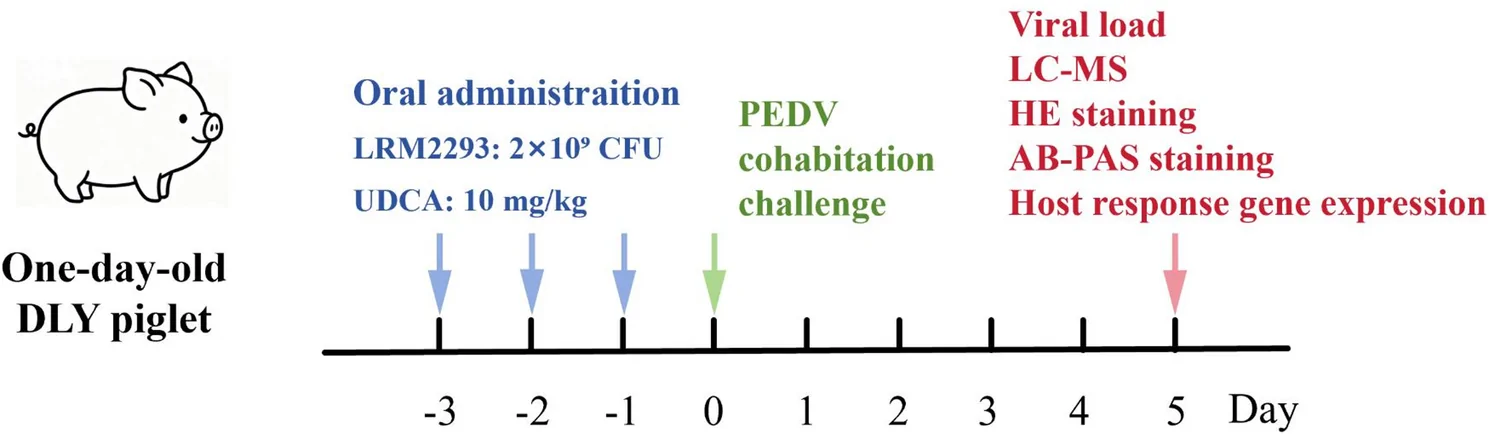
Animal experimental procedure and sample collection. PEDV-negative piglets were pretreated with LRM2293 or UDCA for three consecutive days before PEDV-2022-HEB strain challenge. Clinical symptoms were monitored throughout the experimental period, body weight was recorded daily, and fecal swabs were collected every 6 h within 5 days post-infection. All piglets were humanely euthanized five days following infection. Intestinal tissues and colonic contents were collected for subsequent analyses

Fifteen PEDV-negative neonatal DLY piglets were acquired from a Gushi Agriculture & Animal Husbandry Group Co., Ltd. in Harbin, Heilongjiang Province, China. Piglets were housed in independent negative-pressure isolators under standardized environmental conditions. At the end of the experimental period, the piglets were euthanised by intravenous administration of an overdose of sodium pentobarbital at 100 mg/kg body weight. Loss of consciousness was confirmed by the absence of the corneal and pedal withdrawal reflexes and response to painful stimulation. Death was confirmed by the permanent cessation of spontaneous respiration and heartbeat. Gross pathological examinations were then performed, and jejunal and ileal tissue samples were collected immediately. This procedure was selected to minimise animal pain and distress and to facilitate prompt tissue collection. All procedures were conducted in accordance with the protocol approved by the Animal Care and Use Committee of Northeast Agricultural University (approval number: NEAUEC20220317). The subsequent section details the animal experimental procedures (Fig. 2).

Piglets were randomly assigned to five experimental groups: Mock group, PEDV group, LRM2293 group, UDCA group, Cohabitation infection group. The LRM2293 group received oral administration of 2 mL bacterial suspension (1 × 10^9^ CFU/mL) once daily for three consecutive days before PEDV challenge. The UDCA group received oral administration of UDCA (10 mg/kg body weight) for three consecutive days. Control groups received sterile PBS under identical conditions. On day 4, piglets in the cohabitation infection group were orally inoculated with PEDV HEB strain (1.5 × 10^6^ TCID_50_). Subsequently, infected piglets were co-housed with piglets from the treatment groups to establish a natural transmission model. Clinical manifestations were monitored throughout the experimental period and evaluated qualitatively. Body weight and fecal viral shedding were also monitored.

### Histopathological analysis

For histological evaluation, intestinal tissues were fixed in 4% paraformaldehyde, embedded in paraffin, sectioned, and stained with hematoxylin and eosin (HE) [34]. Morphological measurements were performed using ImageJ software. Ten randomly selected, non-overlapping villi per section were analyzed. The villus height to crypt depth ratio was measured to evaluate intestinal mucosa integrity.

### Alcian Blue-Periodic Acid Schiff (AB-PAS) staining

Tissues fixed in Carnoy’s fixative were paraffin-embedded, sectioned at 4 μm thickness, and stained with Alcian Blue (pH 2.5) followed by Periodic Acid–Schiff (PAS) staining according to a standard protocol [35]. ImageJ software was used for quantitative analysis of goblet cell numbers and intestinal mucus layer thickness. Goblet cell counts were performed on three non-consecutive sections per animal, with five randomly selected crypt-villus units analyzed per section. Goblet cell numbers were expressed per 100 intestinal epithelial cells. Mucus layer thickness was measured at five randomly selected sites per section.

### Statistical analysis

Data are presented as mean ± standard deviation (SD). Statistical analyses were performed using GraphPad Prism 9.0. Differences among multiple groups were evaluated using one-way analysis of variance (ANOVA) followed by Tukey’s post hoc test. An unpaired two-tailed Student’s t-test was used to compare the two groups. All experiments were performed using at least three independent biological replicates. Statistically significant p-values were indicated as * *P* < 0.05, ** *P* < 0.01, *** *P* < 0.001, **** *P* < 0.0001. A *P*-value > 0.05 was labeled as ‘ns’ for no significant difference.

### Figure preparation

Schematic flowcharts were preliminarily drafted using ChatGPT-4o according to the experimental scheme designed by the authors. All scientific content and graphical details were manually checked, modified and confirmed by the authors prior to submission.

## Results

### Comparative analysis of the biotransformation capacity of CDCA to UDCA by *L. reuteri* strains

**Fig. 3:**
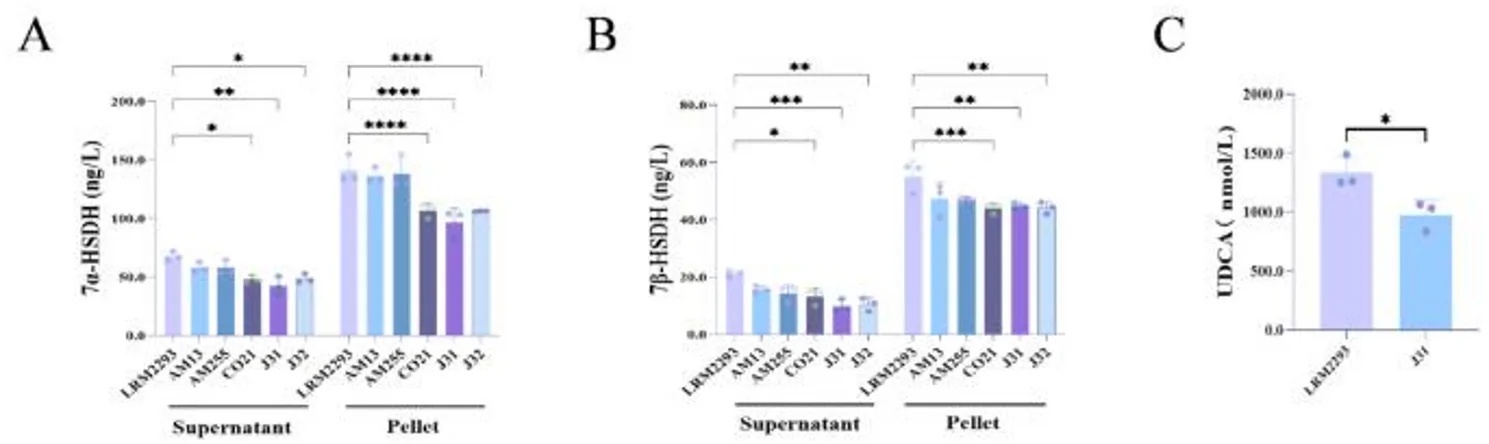
Characterization of CDCA to UDCA biotransformation by *L. reuteri* strains. Enzymatic activities of 7α-HSDH (**A**) and 7β-HSDH (**B**) were measured, and UDCA concentrations in CDCA-supplemented culture supernatants were quantified by LC-MS (**C**). Data are shown as mean ± SD. * P < 0.05, ** *P* < 0.01, *** *P* < 0.001, and **** *P* < 0.0001

To evaluate the biotransformation capacity of CDCA to UDCA, the activities of 7α-HSDH and 7β-HSDH were quantified in both bacterial supernatants and cell pellets of six *L. reuteri* strains isolated from pigs. LRM2293 derived from Min pigs demonstrated higher activities of 7α-HSDH (Fig. 3A) and 7β-HSDH (Fig. 3B) in bacterial supernatants and pellets compared to those derived from DLY pigs (CO21, J31, J32) (*P* < 0.05 for each). LC-MS analysis further showed significantly higher UDCA levels in the CDCA-supplemented culture supernatant of LRM2293 than in that of J31 (*P* < 0.05) (Fig. 3C); values were not baseline-corrected because basal-medium controls were not included.

### Comparison of the anti-PEDV activity of CDCA-transformed supernatants from different *L. reuteri* strains

**Fig. 4:**
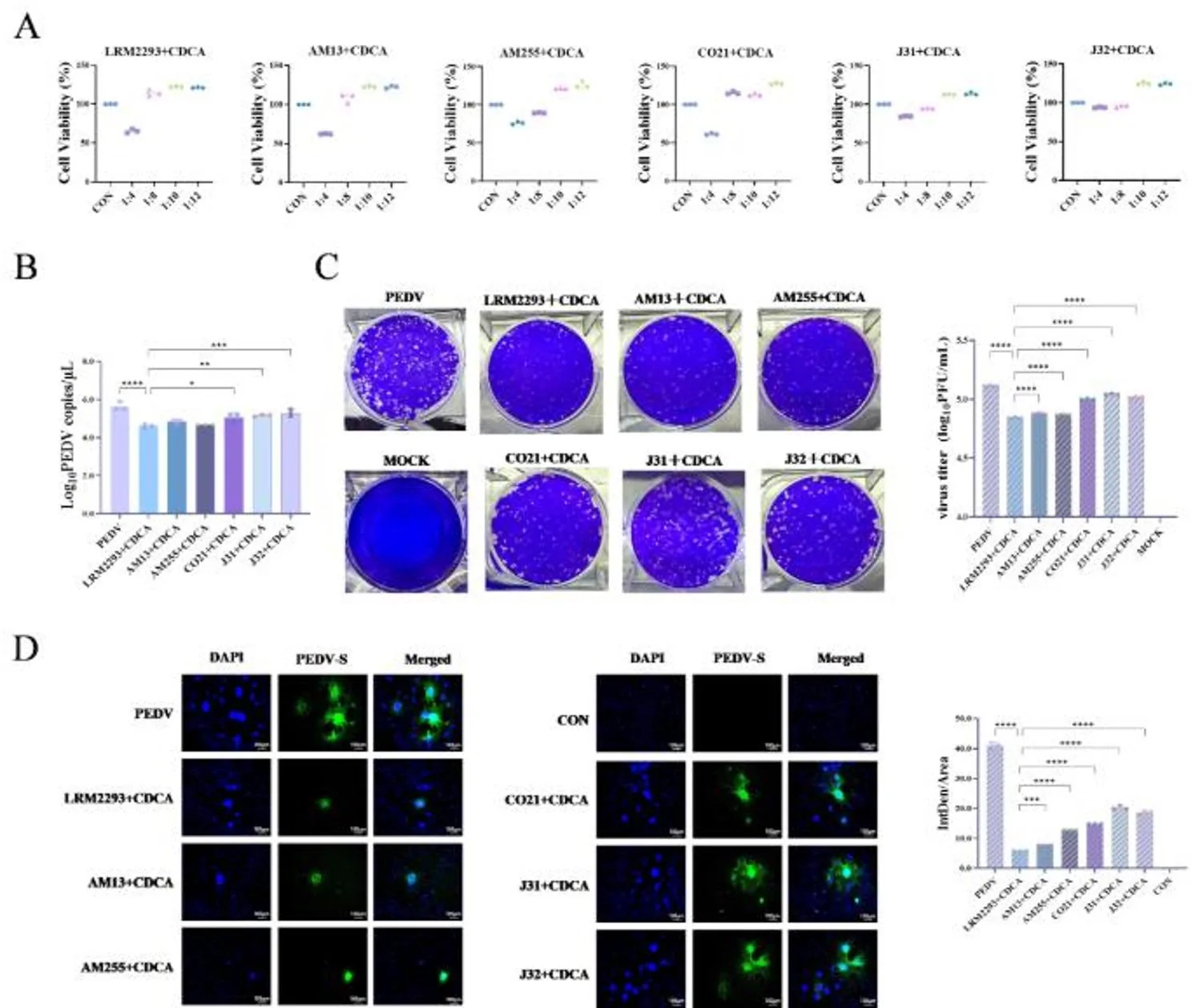
Anti-PEDV activity of *L. reuteri*-transformed CDCA supernatants in IPI-2I cells. IPI-2I cells were infected with PEDV HEB (MOI = 0.1) and treated with non-cytotoxic dilutions of CDCA-transformed supernatants. (**A**) Cytotoxicity assessment by cell viability assay. Antiviral effects were assessed by measuring relative PEDV N mRNA levels (**B**), plaque assay determination of infectious viral titers (**C**), and immunofluorescence staining of PEDV S protein (green) with DAPI-labelled nuclei (blue) (scale bar, 100 μm) (**D**). Data are shown as mean ± SD. * P < 0.05, ** *P* < 0.01, *** *P* < 0.001, and **** *P* < 0.0001

To explore whether bile acid biotransformation capacity was associated with antiviral activity, the anti-PEDV effects of CDCA-transformed supernatants derived from different *L. reuteri* strains were evaluated in IPI-2I cells. The maximum non-toxic dilutions of CDCA-transformed supernatants for Min pig-derived *L. reuteri* strains (LRM2293 + CDCA 1:8; AM13 + CDCA 1:8; AM255 + CDCA 1:10) and DLY-derived strains (CO21 + CDCA 1:8; J31 + CDCA 1:10; J32 + CDCA 1:10) are shown in Fig. 4A. Subsequently, antiviral activity was assessed by RT-qPCR and plaque assays. Compared with DLY pig-derived strains, CDCA-transformed supernatants from Min pig-derived strains exhibited significantly stronger inhibitory effects on PEDV replication (Fig. 4B and C). LRM2293 displayed the greatest antiviral activity, as evidenced by lower viral RNA levels and reduced viral titers. IFA analysis further demonstrated that treatment with LRM2293-transformed supernatant markedly reduced PEDV-associated fluorescence signals and syncytium formation compared with the PEDV control group (Fig. 4D).

The results indicated that min pig-derived *L. reuteri* strains, especially LRM2293, show stronger anti-PEDV activity via CDCA-transformed metabolites.

### Anti-PEDV activity of UDCA in vitro

**Fig. 5:**
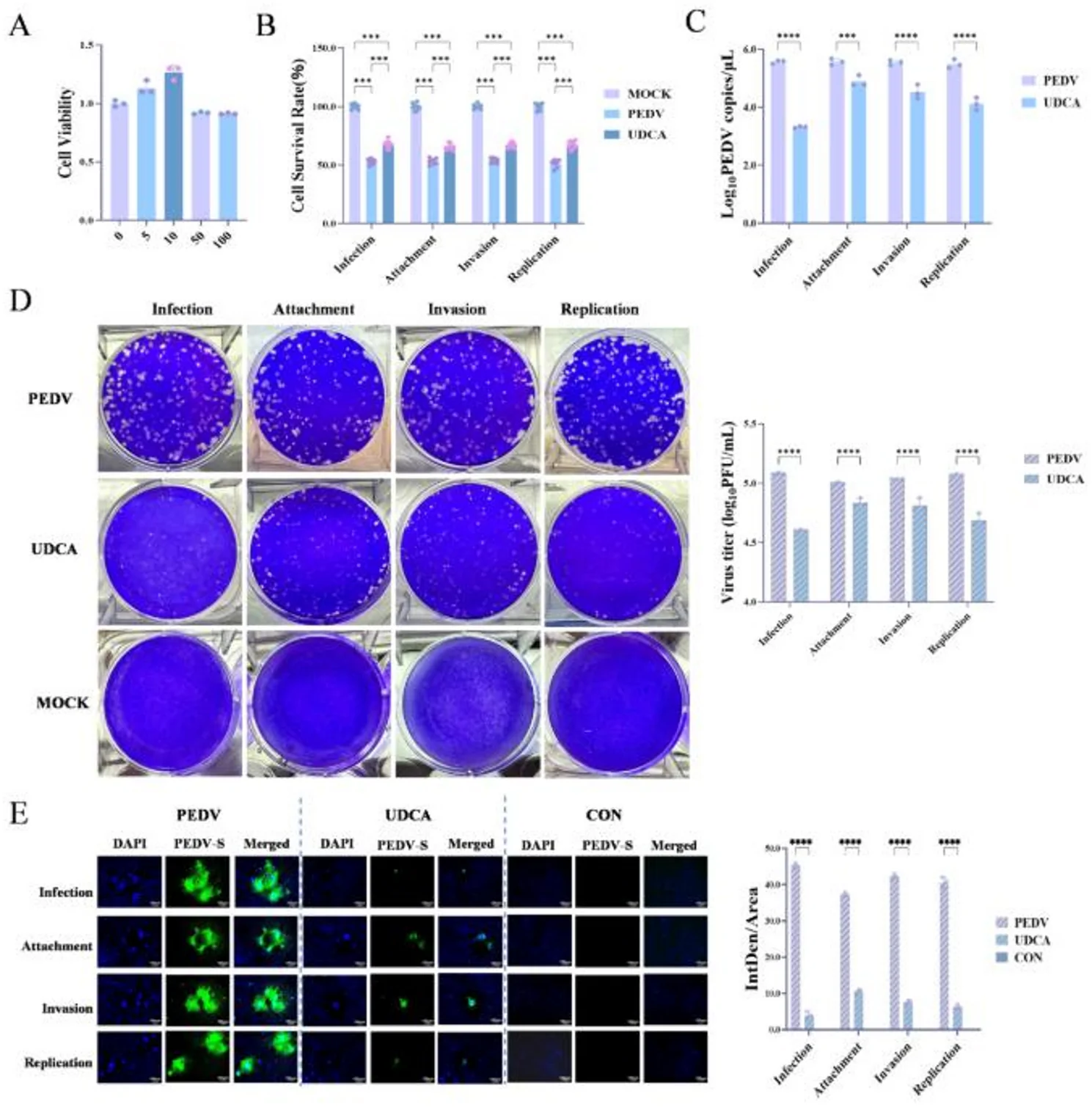
Stage-specific antiviral effects of UDCA against PEDV in IPI-2I cells. IPI-2I cells were infected with PEDV HEB (MOI = 0.1) and treated with UDCA (10 µM) during viral attachment (0–1 h), invasion (1–2 h), replication (2–24 h), or the entire infection period. (**A**) Cytotoxicity assessment by cell viability assay. Antiviral effects were evaluated by cell viability (**B**), relative PEDV N mRNA levels (**C**), infectious viral titers via plaque assay (**D**), and immunofluorescence staining (**E**). PEDV S is shown in green, and nuclei in blue (DAPI) (scale bar, 100 *µ*m). Data are presented as mean ± SD. *** *P* < 0.001, and **** *P* < 0.0001

To further evaluate the antiviral activity of UDCA, its inhibitory effects on various stages of the viral life cycle were investigated. Based on the CCK-8 assay, a non-cytotoxic concentration of 10 µM UDCA was chosen for subsequent experiments (Fig. 5A). Compared with the PEDV-infected group, UDCA treatment significantly improved cell viability at multiple stages of infection (Fig. 5B). RT-qPCR and plaque assay analyses demonstrated that UDCA significantly reduced PEDV RNA levels and infectious viral titers during viral attachment, invasion, and replication (Fig. 5C and D). Among these stages, the most pronounced inhibition was observed during viral replication. Consistent with these findings, IFA analysis showed that UDCA treatment markedly decreased PEDV-associated fluorescence intensity and alleviated virus-induced cytopathic effects (Fig. 5E).

Collectively, these results indicated that UDCA exhibits antiviral activity against PEDV in vitro and may interfere with multiple stages of the viral life cycle, particularly the replication phase.

### LRM2293 and UDCA alleviate PEDV-induced barrier dysfunction, oxidative stress, inflammation, and ER stress in IPI-2I cells

**Fig. 6:**
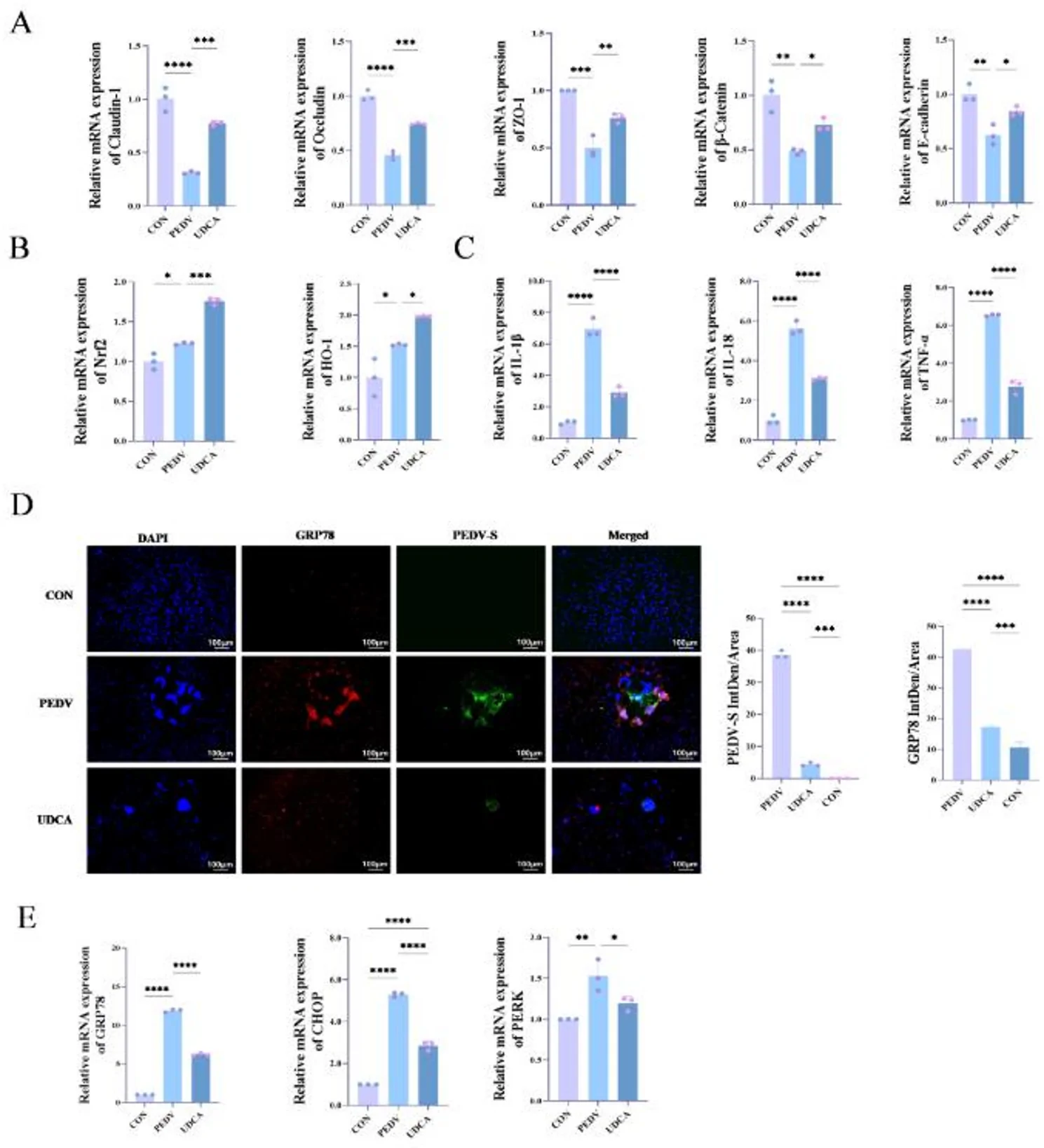
UDCA alleviates PEDV-induced epithelial barrier disruption and cellular stress responses in IPI-2I cells. IPI-2I cells were infected with PEDV HEB (MOI = 0.1) and treated with UDCA (10 µM). Barrier integrity was evaluated by the relative mRNA expression of tight junction and adhesion associated genes (**A**), oxidative stress and inflammatory responses were assessed by antioxidant genes (*Nrf2* and *HO-1*) (**B**) and pro-inflammatory cytokines (*IL-1β*, *IL-18*, and *TNF-α*) (**C**). ER stress was examined by GRP78 immunofluorescence staining (**D**) and relative mRNA expression of *GRP78*, *CHOP*, and *PERK* (**E**). GRP78 is shown in red, PEDV S protein in green, and nuclei in blue (DAPI) (scale bar, 100 *µ*m). Data are presented as the mean ± SD. * P < 0.05, ** *P* < 0.01, *** *P* < 0.001, and **** *P* < 0.0001

PEDV infection disrupted epithelial barrier homeostasis, as reflected by the downregulation of tight junction and adhesion molecules (*Claudin-1*, *Occludin*, *ZO-1*, *β-Catenin*, and *E-Cadherin*; Fig. 6A). Concurrently, PEDV impaired antioxidant defense mechanisms by suppressing *Nrf2* and *HO-1* expression (Fig. 6B). PEDV infection also induced enhanced inflammatory signaling, evidenced by increased expression of *IL-1β*, *IL-18*, and *TNF-α* (Fig. 6C). Moreover, PEDV activated ER stress pathways, resulting in elevated GRP78 fluorescence and increased expression of *GRP78*, *CHOP*, and *PERK* (Fig. 6D-E). UDCA effectively reversed these PEDV-induced alterations, indicating that its antiviral and cytoprotective effects are closely linked to preservation of epithelial barrier function and alleviation of oxidative stress-, inflammation-, and ER stress-associated cellular damage.

### UDCA suppresses PEDV replication in association with TGR5-IFN-λ3 signaling

**Fig. 7:**
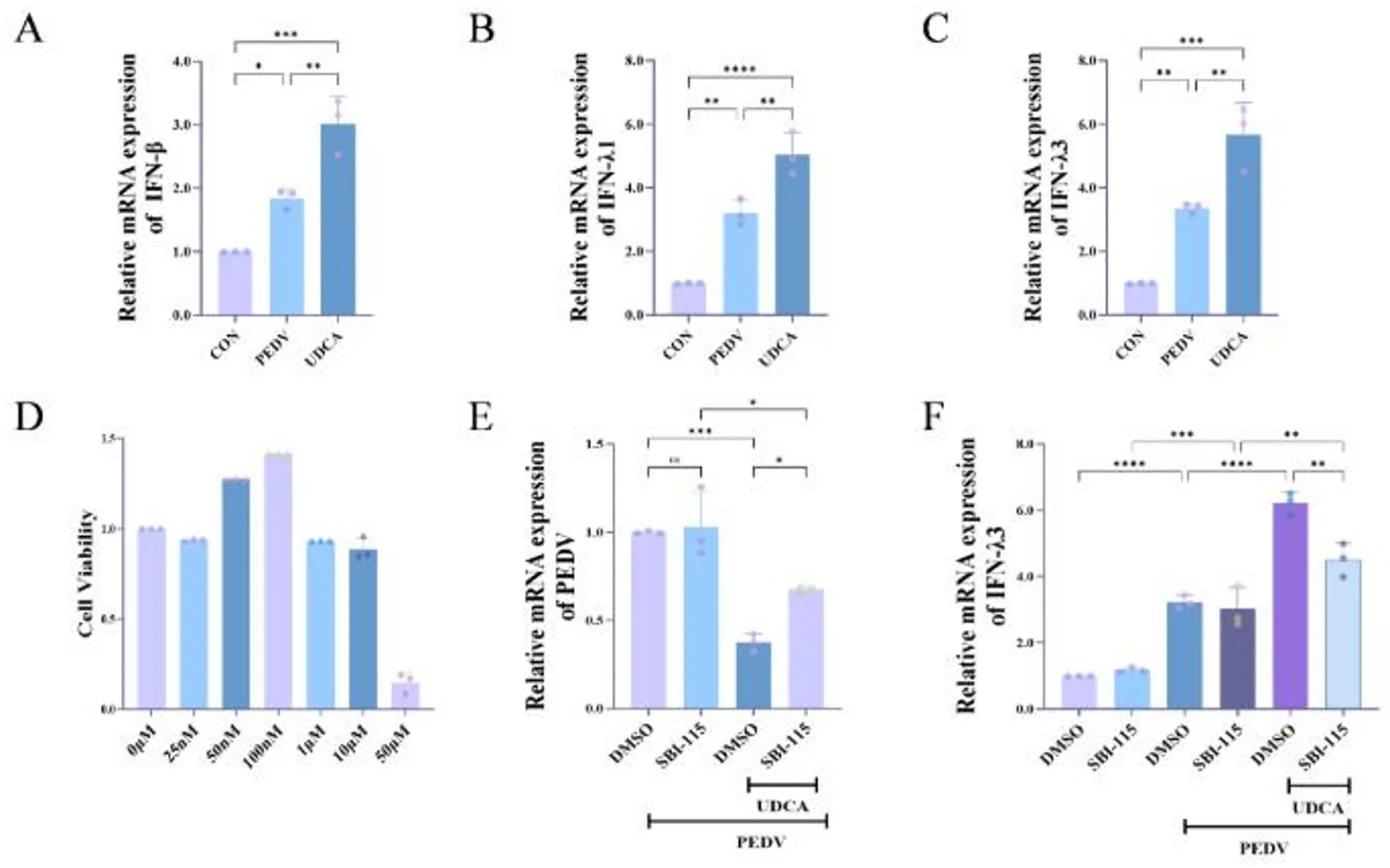
UDCA suppresses PEDV replication associated with TGR5-IFN-λ3 signaling. IPI-2I cells were infected with PEDV HEB (MOI = 0.1) and treated with UDCA (10 µM); SBI-115 (100 nM) was used to inhibit TGR5 where indicated. Interferon responses were assessed by measuring the expression of *IFN-β* (**A**), *IFN-λ1* (**B**), and *IFN-λ3* (**C**). The involvement of TGR5 signaling was further evaluated by examining cell viability (**D**) following treatment with the TGR5 inhibitor SBI-115, relative PEDV N mRNA level (**E**) and relative *IFN-λ3* mRNA level (F). * P < 0.05, ** *P* < 0.01, *** *P* < 0.001, and **** *P* < 0.0001

To investigate potential mechanisms underlying the antiviral activity of UDCA, the expression of interferon-related genes was examined (Fig. 7). PEDV infection induced the expression of *IFN-β*, *IFN-λ1*, and *IFN-λ3* (Fig. 7A-C). Compared with the PEDV group, UDCA treatment further increased the expression of these interferons, with *IFN-λ3* showing the most prominent response (Fig. 7A-C). The TGR5 inhibitor SBI-115 was subsequently used to evaluate the involvement of TGR5 signaling. A concentration of 100 nM SBI-115 was selected based on the absence of detectable cytotoxicity (Fig. 7D). Inhibition of TGR5 partially reversed the reduction in PEDV replication observed following UDCA treatment (Fig. 7E). Similarly, the induction of *IFN-λ3* expression by UDCA was significantly attenuated after SBI-115 treatment (Fig. 7F).

These pharmacological findings support the involvement of TGR5-associated IFN-λ3 signaling in the antiviral activity associated with UDCA; however, the partial reversal indicates that additional TGR5-independent mechanisms may also contribute.

### Protective effects of LRM2293 and UDCA against PEDV infection in piglets

**Fig. 8:**
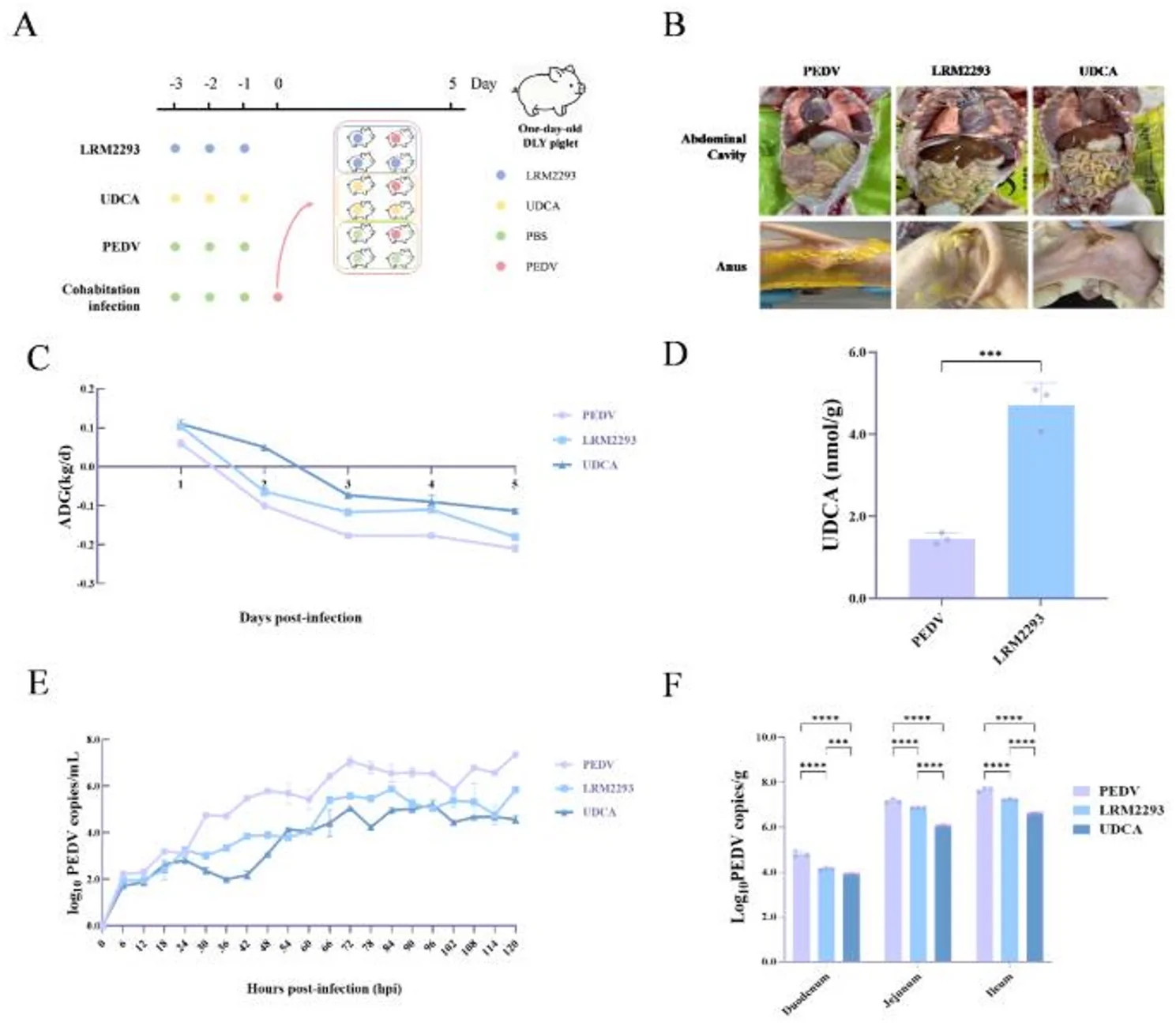
Evaluation of the protective efficacy of LRM2293 and UDCA in PEDV-infected piglets. Piglets were treated with LRM2293 (2 mL, 1 × 10⁹ CFU/mL) or UDCA (10 mg/kg body weight) before PEDV cohabitation challenge. (A) Experimental design. (B) Diarrhea onset was monitored descriptively. (C) Average daily weight gain. (D) Colonic UDCA concentrations quantified by LC-MS. The antiviral efficacy was evaluated by PEDV loads in anal swabs (E) and small intestinal tissues (F). Data are presented as the mean ± SD. *** *P <* 0.001, and **** *P <* 0.0001

To evaluate the protective effects in vivo, neonatal piglets were subjected to a PEDV cohabitation challenge model, as illustrated in the experimental design (Fig. 8A). Piglets in the PEDV group developed diarrhea at approximately 36 h after challenge, accompanied by vomiting and reduced appetite. Diarrhea onset was delayed to approximately 48 h in the LRM2293-treated group and 60 h in the UDCA-treated group, with qualitatively milder clinical manifestations in both treatment groups (Fig. 8B-C). Among the treatment groups, UDCA generally exhibited stronger protective effects. LC-MS analysis revealed significantly increased colonic UDCA concentrations in piglets receiving LRM2293 (Fig. 8D), indicating that this strain influenced intestinal bile acid composition in vivo. Analysis of viral shedding demonstrated that both LRM2293 and UDCA reduced fecal viral loads compared with the PEDV group (Fig. 8E). Consistent with these observations, viral RNA levels in the duodenum, jejunum, and ileum were significantly lower in treated animals (Fig. 8F).

Together, these findings suggested that both LRM2293 and UDCA contribute to host protection against PEDV infection and that modulation of intestinal bile acid metabolism may be associated with this protective effect.

### LRM2293 and UDCA improve intestinal barrier integrity in PEDV-Infected piglets

**Fig. 9:**
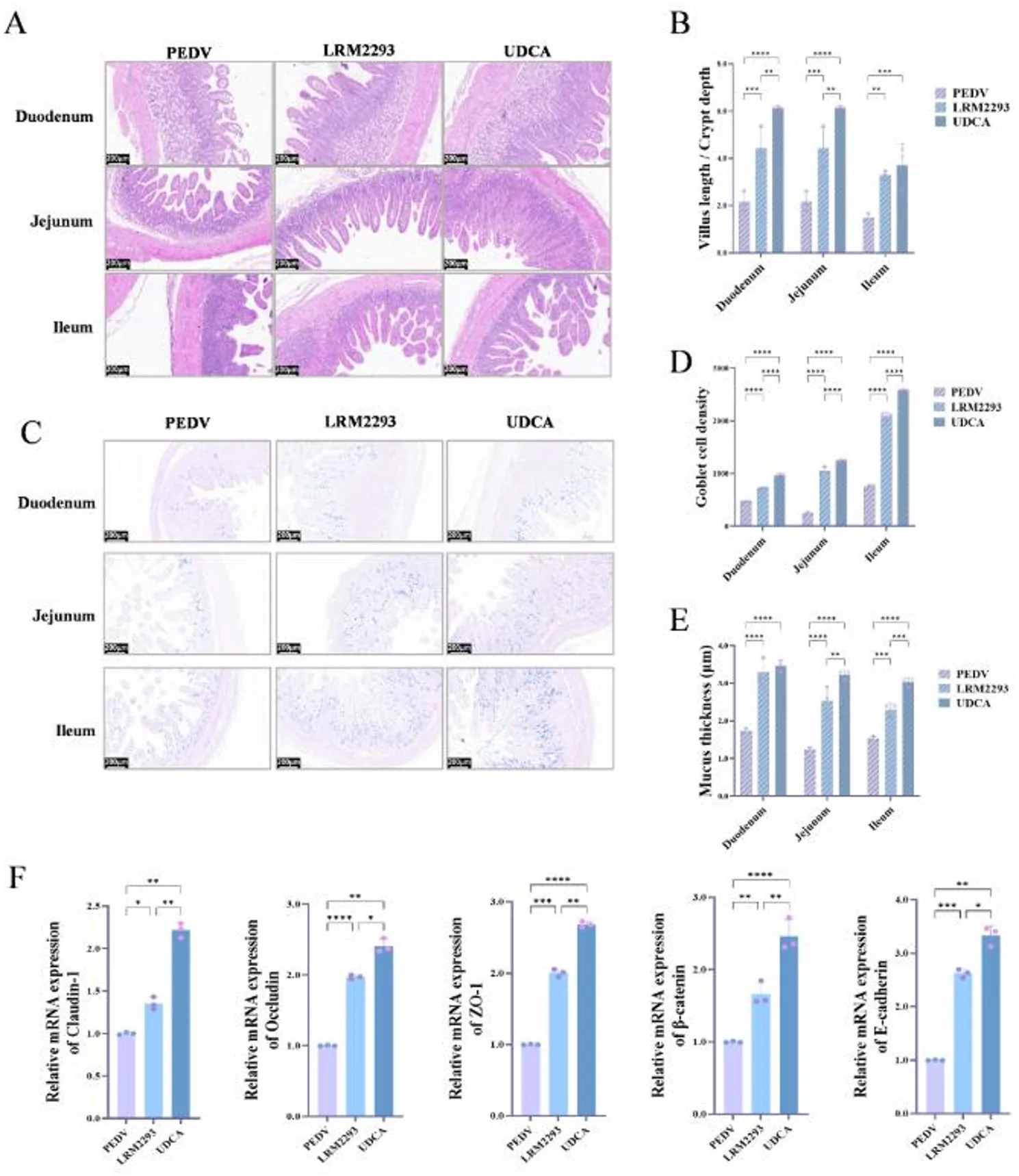
Protective effects of LRM2293 and UDCA on intestinal barrier function in PEDV-infected piglets. Intestinal structural integrity was assessed by H&E staining (A) and villus height to crypt depth ratio (B). Mucosal barrier function was evaluated by AB-PAS staining (C), goblet cell abundance (D), and mucus layer thickness (E). Relative mRNA expression levels of barrier-associated genes, including *Claudin-1*, *Occludin*, *ZO-1*, *β-Catenin*, and *E-Cadherin* (F). Data are presented as the mean ± SD. * *P <* 0.05, ** *P <* 0.01, *** *P <* 0.001, and **** *P <* 0.0001

Histopathological analyses were performed to assess intestinal barrier integrity following PEDV infection. HE staining revealed severe villous atrophy and intestinal mucosal injury in PEDV-infected piglets. In contrast, both LRM2293 and UDCA treatments alleviated intestinal damage and improved villus morphology (Fig. 9A). Quantitative analysis demonstrated significantly increased villus height-to-crypt depth ratios in the treatment groups compared with the PEDV group (Fig. 9B). AB-PAS staining further showed increased goblet cell numbers and mucus layer thickness following treatment with either LRM2293 or UDCA (Fig. 9C-E). Consistent with these histological findings, expression levels of barrier-associated genes, including *Claudin-1*, *Occludin*, *ZO-1*, *β-Catenin*, and *E-Cadherin*, were significantly elevated in treated piglets (Fig. 9F).

Overall, these results suggested that both interventions contribute to maintaining intestinal barrier integrity during PEDV infection, with UDCA showing comparatively stronger effects.

### LRM2293 and UDCA modulate oxidative stress, inflammatory responses, and er stress in PEDV-Infected piglets

**Fig. 10:**
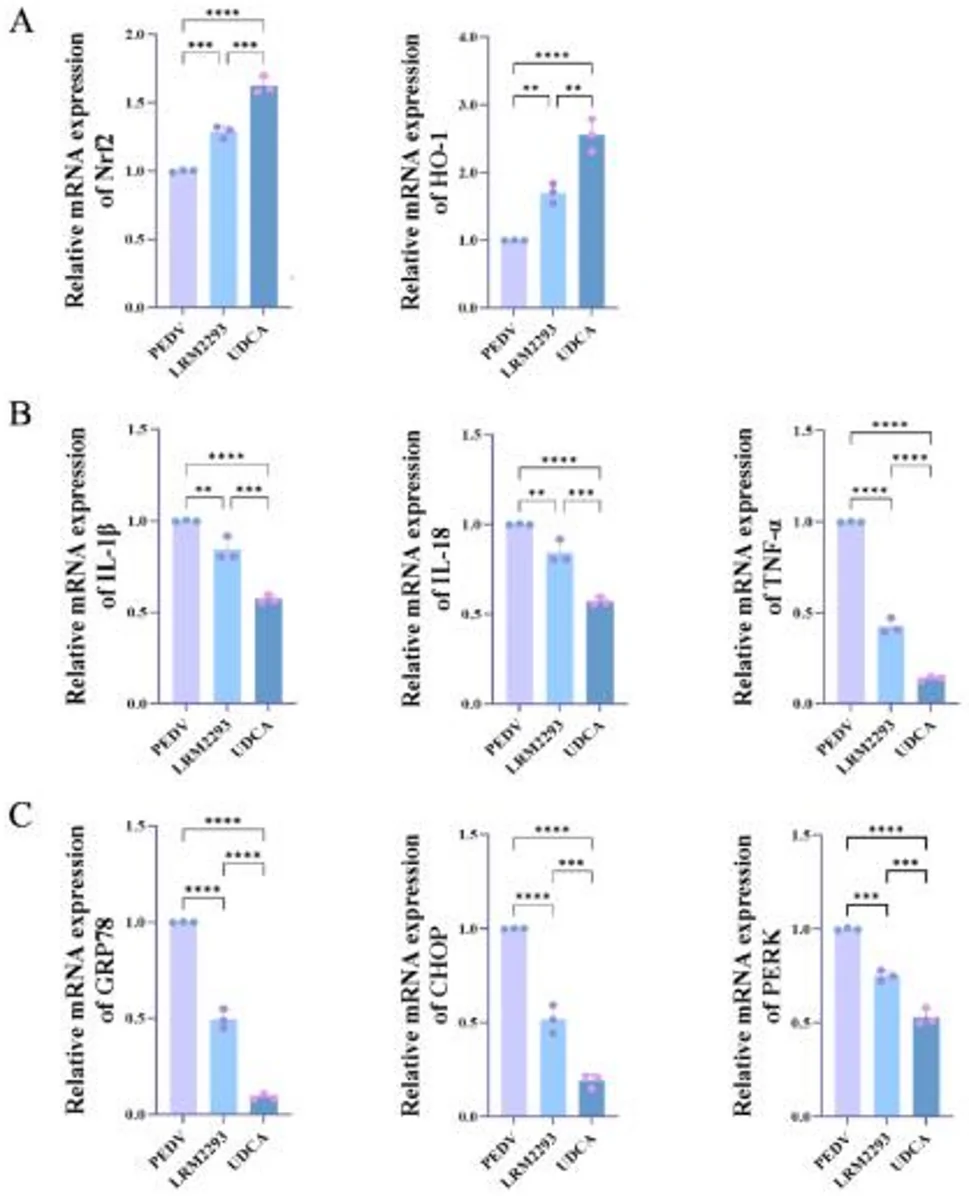
LRM2293 and UDCA alleviate PEDV-induced intestinal oxidative stress, inflammation, and endoplasmic reticulum stress in piglets. **A** Intestinal antioxidant responses were evaluated by measuring relative mRNA levels of *Nrf2* and *HO-1*. **B** Inflammatory responses were assessed by relative mRNA levels *IL-1β*, *IL-18*, and *TNF-α*. **C** Endoplasmic reticulum stress was examined by relative mRNA levels of *GRP78*, *CHOP*, and *PERK*. Data are presented as mean ± SD. ** *P* < 0.01, *** *P* < 0.001, and **** *P* < 0.0001

To further investigate the biological responses associated with protection against PEDV infection, genes related to oxidative stress, inflammation, and ER stress were analyzed in intestinal tissues (Fig. 10). Compared with the PEDV group, treatment with either LRM2293 or UDCA significantly increased the expression of antioxidant-related genes, including *Nrf2* and *HO-1* (Fig. 10A). The expression levels of inflammatory mediators (*IL-1β*, *IL-18*, and *TNF-α*) were significantly reduced in both treatment groups (Fig. 10B). Similarly, the expression of ER stress-related genes (*GRP78*, *CHOP*, and *PERK*) was markedly lower following treatment with LRM2293 or UDCA (Fig. 10C). These observations indicate that the protective effects associated with LRM2293 and UDCA are accompanied by modulation of antioxidant responses, inflammatory signaling, and ER stress pathways. UDCA generally exhibited stronger regulatory effects than LRM2293.

## Discussion

Min pigs are an indigenous Chinese breed characterized by strong disease resistance and environmental adaptability [11, 12]. Previous studies have demonstrated distinct differences in gut microbial composition and metabolic functions between Min pigs and commercial DLY pigs [15, 16]. The gut microbiota and its metabolites serve as crucial regulators of intestinal homeostasis and antiviral immunity [12, 27, 36–38]. Among these metabolites, bile acids function as both digestive molecules and signaling mediators, playing a crucial role in the regulation of immune responses, the maintenance of epithelial barrier integrity, and the modulation of host-microbiota interactions [37, 38]. Our previous metabolomic analysis indicated increased intestinal concentrations of ursodeoxycholic acid (UDCA) in Min pigs, implying an enhanced capacity for microbial bile acid biotransformation [15]. *L. reuteri* is a prevalent probiotic species whose strains exhibit bile acid transformation activity [39, 40]. Certain *L. reuteri* strains demonstrated the ability to efficiently convert primary bile acids into UDCA [41, 42]. However, this functional capability varies considerably among different strains and host species.

PEDV poses a significant threat to the global swine industry [1–3]. Since 2010, highly pathogenic genotype 2 (G2) strains have predominated in outbreaks. The G2b subtype is the most prevalent and responsible for elevated mortality in neonatal piglets, weakening the effectiveness of existing vaccines [8–10]. There is an urgent need for new interventions for bolstering intestinal defense mechanisms. Therefore, we investigated whether Min pig-derived *L. reuteri* strains possess distinct bile acid metabolic characteristics and whether these characteristics are associated with resistance to PEDV infection.

7α-HSDH and 7β-HSDH are involved in the conversion of CDCA to UDCA, significantly impacting UDCA production efficiency [8]. 7β-HSDH activity has previously been identified and biochemically characterized in *Lactobacillus spicheri*, supporting the biological plausibility of HSDH activity in Lactobacillus species [42, 43].

In this study, strain LRM2293 exhibited significantly higher 7α-HSDH and 7β-HSDH activities than DLY-derived strains. Consistently, LC-MS analysis showed higher measured UDCA concentrations in the CDCA-supplemented supernatant of LRM2293 than in that of J31. The stronger anti-PEDV effects observed for Min pig-derived strains occurred alongside higher 7α/β-HSDH activities [44]. Together, these findings are consistent with bile acid remodeling as one potential component of the protective phenotype observed in Min pig-derived strains. However, because the bacterial culture supernatant contains multiple metabolites with potential antiviral activity [45, 46], the present data do not establish a direct correlation between UDCA production and antiviral activity, and other bacterial-derived or CDCA-transformed metabolites may also contribute to the observed antiviral phenotype.

Growing evidence indicates that bile acids participate in antiviral defense through the modulation of host signaling pathways [27–29, 47]. Consistent with these observations, our data revealed that exogenous UDCA significantly inhibited PEDV replication in intestinal epithelial cells. Interestingly, inhibition was observed during viral attachment, invasion, and replication stages, with the strongest effect occurring during viral replication. These findings suggested that UDCA may interfere with multiple phases of the viral life cycle, although the precise molecular targets require further investigation. PEDV infection causes severe disruption of intestinal epithelial integrity, resulting in villous atrophy, increased permeability, inflammatory responses, oxidative stress, and endoplasmic reticulum stress [4–7]. The integrity of the intestinal barrier is crucial for limiting viral infections [13, 48, 49]. The compromise of the intestinal mucosal barrier not only facilitates the attachment and invasion of PEDV into intestinal epithelial cells but also exacerbates systemic inflammatory responses and viral dissemination [50–53]. In the present study, UDCA significantly increased the expression of tight junction proteins and adhesion molecules, including *Claudin-1*, *Occludin*, *ZO-1*, *β-Catenin*, and *E-Cadherin*. Simultaneously, UDCA reduced the expression of pro-inflammatory cytokines and ER stress markers while enhancing antioxidant-related signaling pathways. These observations are consistent with previous reports demonstrating the cytoprotective, anti-inflammatory, and antioxidative properties of bile acid [27, 28].

Interferon-mediated innate immunity represents a central defense mechanism against viral infection [54, 55]. IFN-λ3 preferentially targets mucosal epithelial cells, playing a crucial role in intestinal antiviral defense while minimizing excessive systemic inflammation [56, 57]. Bile acid receptor TGR5 can enhance antiviral innate immunity by interferon signaling [58]. This study found that UDCA elevated the expression of IFN-β, IFN-λ1, and especially IFN-λ3 in cells infected with PEDV. Pharmacological inhibition of TGR5 partially reversed both IFN-λ3 induction and the antiviral activity of UDCA, supporting the potential involvement of TGR5-associated IFN-λ3 signaling in the antiviral response to UDCA [27]. However, these pharmacological data primarily demonstrate an association and do not establish a definitive causal relationship between TGR5 activation, IFN-λ3 induction, and the antiviral effects of UDCA. In particular, the partial reversal observed following TGR5 inhibition suggests that additional TGR5-independent mechanisms may also contribute to UDCA-mediated antiviral activity. Therefore, the TGR5–IFN-λ3 axis should be considered a potential signaling mechanism associated with the antiviral effects of UDCA rather than an exclusively established causal pathway. Further studies using genetic approaches, such as TGR5 knockdown or knockout, together with direct manipulation of IFN-λ3 signaling, will be required to establish the causal contribution of this pathway. In addition, TGR5-associated IFN-λ3 signaling was not evaluated in intestinal tissues from the piglet experiment, and further in vivo validation is necessary.

The PEDV HEB strain utilized in this study is classified within the GIIb genotype and demonstrates considerable pathogenicity in neonatal piglets [13]. To assess the protective efficacy of LRM2293 and UDCA against PEDV in piglets, a cohabitation challenge experiment was conducted. Oral administration of either LRM2293 or UDCA delayed diarrhea onset, reduced viral shedding, improved growth performance, and alleviated intestinal pathology. Histological analysis further demonstrated preservation of villus architecture, increased goblet cell numbers, and enhanced mucus layer thickness. These findings indicate that both interventions contribute to maintaining intestinal barrier integrity during PEDV infection. Oral administration of LRM2293 resulted in increased colonic UDCA concentrations, supporting the ability of this strain to modulate the intestinal bile acid environment in vivo. Together with the enhanced CDCA-to-UDCA biotransformation observed in vitro and the protective effects of exogenous UDCA, these findings suggest an association between LRM2293-mediated bile acid remodeling and resistance to PEDV infection. This interpretation is also consistent with previous evidence showing that bile acid remodeling and bile acid-mediated signaling can modulate host antiviral responses [27, 44]. However, the present study does not establish UDCA as the sole or predominant mediator of LRM2293-mediated protection. Other bacterial-derived metabolites, probiotic-mediated modulation of intestinal barrier function and host immune responses, and broader microbiota–host interactions may also contribute to the observed antiviral phenotype [13, 14]. Therefore, the protective effects of LRM2293 are likely multifactorial, with bile acid remodeling representing one potentially important component. Further studies using targeted approaches to specifically manipulate UDCA production will be required to determine the precise contribution of UDCA to LRM2293-mediated antiviral protection. A notable observation in this study was that direct UDCA supplementation produced stronger antiviral and protective effects than administration of LRM2293 alone. This finding may reflect differences in bioavailability, metabolic efficiency, or the time required for microbial colonization and metabolite production. The superior efficacy observed for UDCA suggests that direct postbiotic intervention may represent a promising complementary strategy to conventional probiotic administration. The Min pig origin and enhanced bile acid biotransformation capacity of LRM2293 highlight the potential value of indigenous pig-derived probiotics with distinctive metabolic functions [13, 14]. LRM2293 may therefore represent a promising probiotic candidate for PEDV control. However, its efficacy requires further validation under commercial farm conditions, where environmental, management, microbiota, and viral factors are more complex. Further validation utilizing genetically diverse PEDV strains is necessary.

Overall, the present study provides evidence that Min pig-derived LRM2293 possesses enhanced bile acid biotransformation capacity and is associated with increased UDCA levels. Both LRM2293 and UDCA exhibited protective activity against PEDV infection in vitro and in vivo. These findings suggested that bile acid remodeling may represent one component of the protective effects associated with LRM2293 and support further investigation of the gut microbiota–bile acid axis in PEDV prevention and control.

## Data Availability

The datasets generated and/or analysed during the current study are available from the corresponding author on reasonable request.
